# Dynamics of the Peripheral Membrane Protein P2 from Human Myelin Measured by Neutron Scattering—A Comparison between Wild-Type Protein and a Hinge Mutant

**DOI:** 10.1371/journal.pone.0128954

**Published:** 2015-06-11

**Authors:** Saara Laulumaa, Tuomo Nieminen, Mari Lehtimäki, Shweta Aggarwal, Mikael Simons, Michael M. Koza, Ilpo Vattulainen, Petri Kursula, Francesca Natali

**Affiliations:** 1 Biochemistry and Molecular Medicine & Biocenter Oulu, University of Oulu, Oulu, Finland; 2 German Electron Synchrotron (DESY), Hamburg, Germany; 3 European Spallation Source (ESS), Lund, Sweden; 4 Department of Physics, Tampere University of Technology, Tampere, Finland; 5 Max Planck Institute for Experimental Medicine, Göttingen, Germany; 6 Institut Laue-Langevin (ILL), Grenoble, France; 7 Department of Biomedicine, University of Bergen, Bergen, Norway; 8 CNR-IOM, OGG, Grenoble, France; Jacobs University Bremen, GERMANY

## Abstract

Myelin protein P2 is a fatty acid-binding structural component of the myelin sheath in the peripheral nervous system, and its function is related to its membrane binding capacity. Here, the link between P2 protein dynamics and structure and function was studied using elastic incoherent neutron scattering (EINS). The P38G mutation, at the hinge between the β barrel and the α-helical lid, increased the lipid stacking capacity of human P2 *in vitro*, and the mutated protein was also functional in cultured cells. The P38G mutation did not change the overall structure of the protein. For a deeper insight into P2 structure-function relationships, information on protein dynamics in the 10 ps to 1 ns time scale was obtained using EINS. Values of mean square displacements mainly from protein H atoms were extracted for wild-type P2 and the P38G mutant and compared. Our results show that at physiological temperatures, the P38G mutant is more dynamic than the wild-type P2 protein, especially on a slow 1-ns time scale. Molecular dynamics simulations confirmed the enhanced dynamics of the mutant variant, especially within the portal region in the presence of bound fatty acid. The increased softness of the hinge mutant of human myelin P2 protein is likely related to an enhanced flexibility of the portal region of this fatty acid-binding protein, as well as to its interactions with the lipid bilayer surface requiring conformational adaptations.

## Introduction

In the vertebrate central (CNS) and peripheral nervous systems (PNS), selected neuronal axons are covered by a protecting layer of myelin. The myelin sheath is a unique multilayered membrane, wrapped around PNS axons by Schwann cells. Myelin in the CNS is made by oligodendrocytes. Myelin enables the rapid saltatory conduction of nerve impulses along axons, which can be up to a meter long in the PNS [1]. The myelin membrane contains 75–80% lipids by dry weight, with a high, 30–40%, cholesterol content [2], and it is enriched in myelin-specific proteins that are expressed by myelinating cells [3,4]. The myelin proteome has been under investigation for decades [5,6], but the structure and function of several myelin proteins are still poorly understood [3].

**Fig 1:**
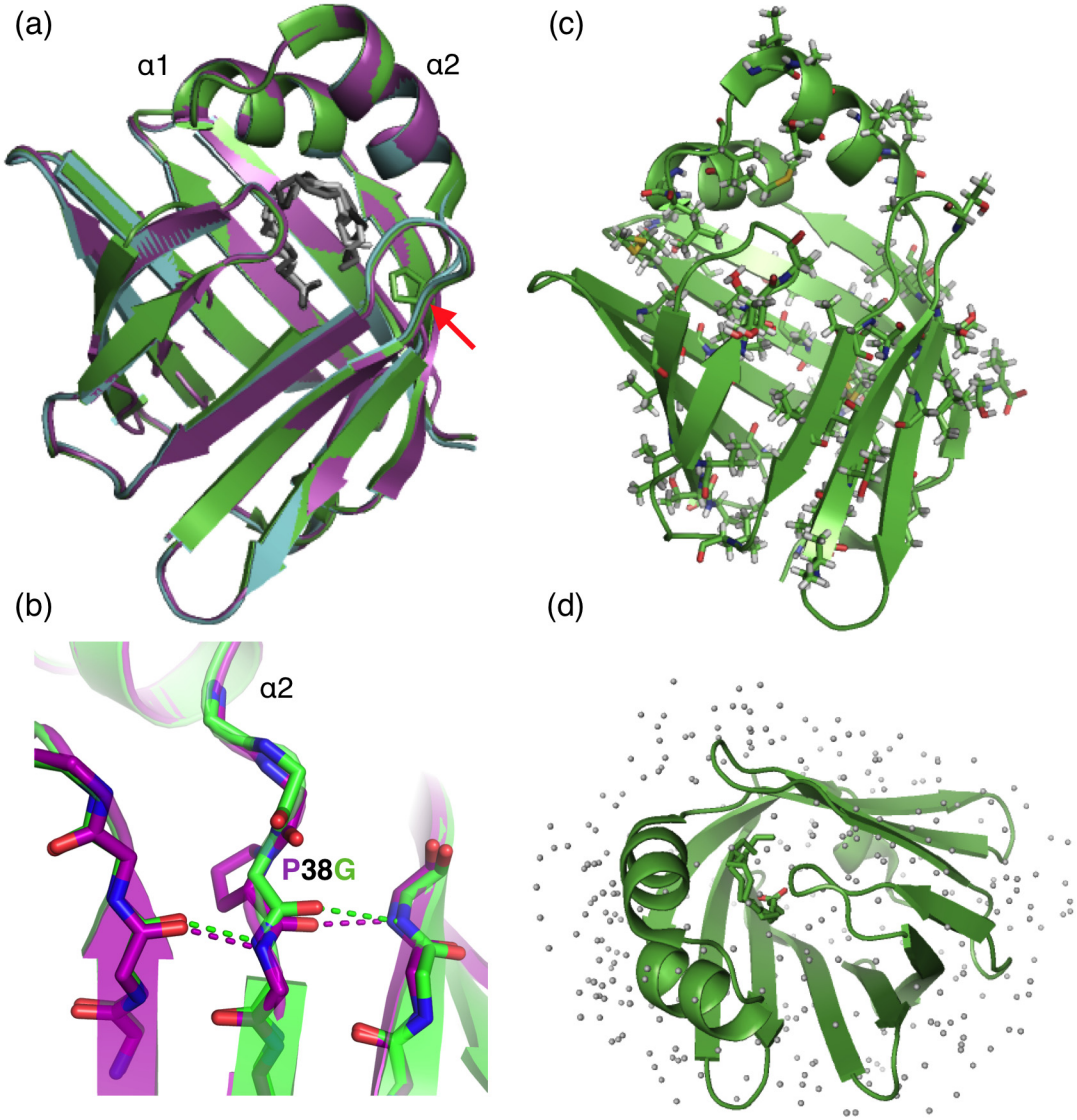
Crystal structure of human P2. (a) Superimposed structures of human wtP2 (cyan) and P2-P38G (green) before neutron scattering experiments and wtP2 after the experiment (magenta). Pro38 is indicated with the red arrow. (b) The P38G mutation site. Note how the main-chain hydrogen bonding (dashed lines) remains conserved at the edge of the β sheet also in the mutant. (c) Locations of the residues with side-chain methyl groups in P2 are shown as sticks. (d) A top view of the barrel-shaped P2 with bound fatty acid and stationary water molecules at cryo temperatures in the high-resolution crystal structure [11].

Myelin protein P2 is one of the quantitatively major proteins of human peripheral nerve myelin. P2 has been reported to comprise up to 15% of total myelin protein [7]; however, it is only present in selected myelin sheaths [8,9]. The crystal structure of human P2 [10] has been solved at atomic (0.93 Å) resolution: 10 antiparallel β strands form a barrel, which is covered by a lid-like motif formed of two α helices (Fig 1) [11]. P2 belongs to the fatty acid binding protein (FABP) family; it has the ability to bind lipids and possibly plays a role in lipid transport and homeostasis in myelin, as indicated by the phenotype of P2-deficient mice [9]. While myelin appears normal in these animals, they have decreased nerve conduction velocity and an altered myelin lipid composition during development [9]. The β barrel of P2 contains a large (1000 Å^3^) ligand-binding pocket that is filled with a fatty acid and water molecules in the crystal structure [10]. Among myelin lipids, one natural ligand for P2 could be cholesterol, which is present in myelin at high concentrations and would fit into the cavity by size and by its electrostatic properties [10].

P2 binds phospholipids and stacks lipid bilayers together [11,12]. We previously reported decreased overall proton dynamics in liposomes in solution at physiological temperatures upon the addition of the P2 protein [13], as well as effects of bound P2 on multilayered lipid membrane structure [14]. P2 has been suggested to stack membrane layers together by binding to phospholipids through its positively charged surface residues, hydrophobic side chains from the α-helical lid partially penetrating the membrane [10,11]. Despite detailed mapping of the structural properties and physiological activity of the P2 protein, its role in healthy and diseased myelin remains unclear.

An insight into molecular motions and flexibility is important for understanding structure-function relationships in proteins [15,16]. Elastic incoherent neutron scattering (EINS) is a powerful tool for investigating quantitative protein dynamics [17–20]. EINS allows inspection of mean square displacements (MSD) of atoms as a function of external parameters, such as temperature, revealing information about eventual transitions occurring in proteins and the force constants required for protein motion activation. Using cold and thermal neutrons with energy resolutions from 70 to 0.9 μeV, atomic fluctuations on the time scale from 10 ps to 1 ns can be observed.

The effects of point mutations on protein dynamics have been studied using EINS before. When a mutation led to a nonfunctional enzyme or partial unfolding of the protein, the flexibility of the protein increased [21,22]. We previously reported an initial characterisation of the properties of human peripheral myelin protein P2 by studying protein dynamics on a fast dynamics scale (10 ps) using EINS [23]. We used wild-type P2 protein (wtP2) and its proline-38-to-glycine (P2-P38G) point mutant to investigate the dynamics of pure proteins as hydrated powders. P2-P38G showed increasing dynamics compared to wtP2 above 220 K [23]. Pro38 is a conserved residue located at a predicted hinge region, assumed to be involved in the opening of the helical lid of P2 upon ligand binding and release [11].

The aim of the present work was to extend earlier investigations on human P2 dynamics [23], expanding the time scale to 100 ps and 1 ns. We also carried out a thorough structural and functional characterisation of the samples to link the observed differences in dynamics to biomolecular function, and to confirm sample stability during the lengthy neutron scattering experiments. The results indicate that using EINS, we can obtain functionally relevant information on protein proton dynamics and the differences therein between the wild-type protein and a functional mutant.

## Materials and Methods

### Protein purification and EINS sample preparation

Human myelin wtP2 and P2-P38G [24] were expressed recombinantly in *E*. *coli* Rosetta (DE3) cells. The proteins were purified using Ni-ion affinity and size-exclusion chromatography, as previously described [10,24].

To get rid of buffer and salt traces in the protein sample, the purified protein was dialyzed three times against a large volume of H_2_O. The protein was then lyophilised to remove all the water and rehydrated with heavy water (D_2_O) to *h* = 0.28 g/g (where *h* is defined as g_D2O_/g_protein_) in a dessicator in a N_2_/D_2_O atmosphere. Due to the large excess of solvent deuterium atoms and the long equilibration time, this procedure guaranteed that most of the exchangeable hydrogen atoms in the protein sample were replaced by deuterium. The hydration level was controlled by weighing the sample during the hydration process. To obtain comparable wild-type and mutant protein samples, both samples were prepared in parallel.

### Circular dichroism spectroscopy

Circular dichroism (CD) spectroscopy was used to study the folding and the secondary structure content of the sample proteins in solution. CD spectra were measured in H_2_O at 0.25 mg/ml before and after the neutron scattering experiments using a Chirascan Plus spectropolarimeter (Applied Photophysics, United Kingdom) and a 0.5-mm quartz cuvette. Melting curves were measured from 293 to 363 K with a heating rate of 1 K/min at 0.25 mg/ml, in a buffer containing 0.8 mM HEPES (pH 7.5), 6 mM NaCl, and 0.4% glycerol.

### Protein crystallography

P2-P38G was crystallised by vapour diffusion at 277 K using a well solution containing 3.15 M ammonium sulphate and 100 mM Tris (pH 8.5). X-ray diffraction data were collected at 100 K on beamline X12 at the DORIS storage ring, EMBL-Hamburg/DESY. wtP2 that had been used for neutron scattering experiments was resolubilised in protein buffer (0.8 mM HEPES (pH 7.5), 6 mM NaCl, 0.4% glycerol) and crystallised with vapour diffusion at 281 K, over a well solution of 28% PEG6000 and 0.1 M sodium citrate (pH 5.5). The diffraction experiment was done at 100 K on the I911-3 beamline, MAX-Lab (Lund, Sweden).

All data were processed with XDS [25], and the structures were solved with molecular replacement using wtP2 [10] as the search model. Refinement was carried out in phenix.refine [26] and model building in coot [27]. Molprobity [28] was used for structure validation. The structure factors and final refined coordinates were deposited at the Protein Data Bank with entry codes 4D6A (wtP2 after neutron scattering experiments) and 4D6B (P2-P38G).

### Molecular dynamics simulations

The crystal structures of wtP2 and P2-P38G were subjected to atomistic molecular dynamics simulations, in order to pinpoint possible regions of differential dynamics linked to the mutation. Simulations were carried out in the presence and absence of bound fatty acid, and hydrogen dynamics were specifically addressed in the analysis.

The protein and the palmitate group were modeled by the CHARMM36 force field [29]. The protein model including palmitate was obtained from the PDB entry 4BVM [11] and converted to the CHARMM36 force field. The topology for wt-P2 was obtained directly from the conversion. P2-P38G was constructed from the wild type topology by changing the amino acid at the point of mutation. The three-point TIP3P model was used for water.

Four different protein systems were considered: wt-P2 and the P2-P38G, both with and without palmitate inside the binding pocket. The proteins were added to a solvated simulation box of approximately (8 x 8 x 8) nm^3^ with 16000 water molecules. Ten Cl^-^ ions were included to neutralise the total charge of the protein that had a palmitate in its pocket (in systems without palmitate, the number of Cl^-^ ions was 11 due to the charge of the palmitate).

The simulations were carried out under NpT conditions. Pressure coupling was done using the isothermal Parrinello-Rahman barostat [30] at a reference pressure of 1 bar, with a coupling time constant of 2.0 ps and isothermal compressibility of 4.5 x 10^–5^ bar^-1^. Temperature coupling was done with the velocity-rescale method [31], with separate temperature coupling groups for the protein and the solvent. The reference temperatures were set as 300 K, with coupling time constants of 2.0 ps. Periodic boundary conditions were used. All bonds were constrained with the LINCS algorithm. The cut-off radii for the neighbor list, the Lennard-Jones interactions, and non-bonded interactions were set at 1.0 nm. For long-range electrostatics, we used the Particle-Mesh Ewald (PME) method with cubic interpolation (PME order 4) and a spacing of 0.16 nm for the Fourier grid.

The simulations were conducted using the GROMACS 4.6 simulation package [32]. The time increment used in integrating the equations of motion was 2 fs. The systems were first energy-minimised with the steepest descent algorithm and then simulated for a total of 3 μs each. The systems were allowed to equilibrate for 500 ns, and the remaining part of the trajectory—over a period of 2.5 μs—was used for analysis. The coordinate files for the trajectory were saved at a rate of (50 ps)^-1^, except in simulations used for the calculation of the MSD, see below.

For analysis of MSDs of the protein hydrogens, we used additional simulations, since the dynamics of hydrogen movement are very rapid. As a starting point, we used the full 3-μs trajectories discussed above. After the 500-ns equilibration period, seven different starting structures for each system were taken at 200-ns intervals, to obtain sufficient sampling over the whole simulation period. Each starting structure was then simulated for 3 ns, while saving the system coordinates every 2 fs to make sure that all the information of the rapid hydrogen movement would be accounted for in the analysis of MSDs. The root-mean square (RMS) fluctuation was calculated for each protein residue with the GROMACS tool g_rmsf.

MSDs of the protein hydrogen atoms were calculated to compare hydrogen mobility between the different systems. To this end, hydrogen mobility was explored for two different groups separately: i) hydrogens in CH_3_-groups, and ii) hydrogens in CH_2_- and CH-groups. For analysis (after the simulations had been completed), the protein was centered, and the rotation and translation of the protein backbone were fitted to the center of the simulation box, thus constraining protein movement during the analysis. The MSDs of the hydrogen atoms, compared to the initial starting structures, were then calculated from each of the 3-ns simulations with the GROMACS tool g_msd, using all the data in the trajectories. The final MSD curve for each case was obtained by averaging over the seven separate curves. In the results, we show the MSD behavior up to 1 ns, where the data are most accurate.

### Functional assays

The lipid stacking properties of P2 were measured by following the turbidity resulting from vesicle aggregation, essentially as described [11]. 0–10 μM P2 was mixed with 0.5 mM DMPC/DMPG (1,2-dimyristoyl-sn-glycero-3-phosphocholine/1,2-dimyristoyl-sn-glycero-3-phosphoglycerol) vesicles in 10 mM HEPES pH 7.4, 150 mM NaCl, and turbidity was measured at 600 nm using an Infinite M200 plate reader (Tecan, Switzerland).

In order to follow stacked membrane domain formation induced by P2 in living mammalian cells, a previously established method [11,33] was used. Briefly, wtP2 and P2-P38G were expressed in Ptk2 cells as fusions with green fluorescent protein and a transmembrane domain, and the cellular membrane organisation was followed by fluorescence microscopy. The formation of brightly fluorescent membrane domains is an indication of membrane stacking in this experimental system [33].

### EINS data collection

To investigate protein dynamical properties, temperature-dependent EINS scans were performed on the thermal and cold neutron high resolution backscattering spectrometers IN13 and IN16, respectively, as well as on the time-focusing time-of-flight spectrometer IN6, all located at the Institut Laue-Langevin (ILL), Grenoble, France.

With a nearly Q-independent energy resolution of δE = 8 μeV (full width at half maximum, FWHM) and an accessible momentum transfer range of 0.2 < Q < 4.9 Å^–1^, IN13 allows the investigation of molecular motions on a time scale up to 100 ps and with an amplitude from 1.3 Å to ~ 31 Å [34].

IN16 [35] is situated on a guide looking at one of the cold sources of the ILL. The Si(111) reflection of the monochromator is used to select a wavelength of λ = 6.27 Å from the incoming neutrons. The instrumental setup results in a very narrow elastic energy resolution of 0.9 μeV (FWHM), corresponding to a time window of ≈1 ns and a momentum transfer range of 0.19 < Q < 1.89 Å^-1^.

IN6 was used at an incident wavelength of λ = 5.1 Å, providing an energy resolution of 70 μeV (FWHM) and proton dynamics on the 10-ps time scale. The setting allows to access the momentum transfer range 0.3 < Q < 2 Å^-1^.

In all experiments, neutron scattering spectra were collected over a wide temperature range (20–310 K), with heating rates properly chosen to optimise the signal-to-noise ratio: 0.6 K/min from 20 K to 100 K, 0.4 K/min from 100 K to 220 K, and 0.15 K/min from 220 K to 310 K. Data were then binned using 5-K steps. As the same samples were used for data collection at all the instruments, the temperature scans were limited to 310 K to prevent the proteins from irreversible unfolding. To control sample integrity, protein structure was also analyzed after the neutron scattering experiments (see above).

The program LAMP [36] was used for data correction. The elastic scattered intensities were corrected for the empty cell contribution and normalised with respect to the lowest temperature scan (T = 20 K) to compensate for differences in detector efficiency and geometry. In order to avoid corrections from multiple scattering events, cell thickness and geometry were properly chosen to minimise neutron absorption by the sample. A typical transmission of ∼95% was guaranteed using standard flat aluminium sample holders with a thickness of 0.4 mm. No Bragg peaks from ice were detected in any of the spectra, which excludes the presence of free water in the samples.

### EINS data analysis

Based on the atomic composition of P2, coherent scattering is estimated to correspond to less than 8% of the total scattering. Thus, in the following, it will be neglected, and we will focus exclusively on the behaviour of the incoherent scattering contribution.

The normalised incoherent elastic neutron scattering *S*_*inc*_(*Q*,ω = 0) can be fitted using the Gaussian approximation within the region of its validity [17]:

$$S=I_{0}e^{-\left(\frac{\langle u^{2}\rangle Q^{2}}{6}\right)}\;,$$ (1)

where 〈*u* ^2^〉/6 is the normalised MSD, and 〈*u* ^2^〉 is defined as

$$\langle u{}^{2}\rangle =\langle u_{r}^{2}-u_{20K}^{2}\rangle .$$ (2)

In protein samples, incoherent scattering is dominated by hydrogen atoms, because hydrogen comprises approximately 50% of all protein atoms and the incoherent scattering cross section of hydrogen (80.27 barn) is significantly larger than that of other atoms in biological macromolecules (nitrogen 0.5 barn, carbon and oxygen 0.001 barn) [37]. With a hydration of *h* = 0.28 g/g, the scattering contribution arising from the solvent (D_2_O) is 1% of the total scattering (deuterium: 2.05 barn), which can be neglected.

Proteins are inhomogeneous macromolecules, each having a main chain of defined length and a unique side chain sequence and composition. Thus, one possibility is to describe protein dynamics using a bimodal distribution model [38,39]. In this model, hydrogen atoms are described as two different populations of homogeneously fluctuating atoms: methyl group hydrogen atoms and all other (non-methyl) non-exchangeable hydrogen atoms. Both populations can be described by a Gaussian function with a different width and the bimodal distribution of hydrogen atom MSD by

$$f(\langle u\;{}^{2}\rangle )=a_{1}\delta (\langle u\;{}^{2}\rangle -\langle u\;{{}^{2}\rangle }_{1})+a_{2}\delta (\langle u\;{}^{2}\rangle -\langle u\;{{}^{2}\rangle }_{2}),$$ (3)

where *δ*(*x*) is a delta function, *a*_1_ and *a*_2_ (with *a*_*2*_ = 1–*a*_1_) represent the population fractions (temperature-independent), and 〈*u* ^2^〉_1_ and 〈*u* ^2^〉_2_ are the average MSDs of the hydrogen atoms in each fraction.

Using the Gaussian distribution function, the bimodal scattering function becomes

$$S\left(Q,\omega =0\right)=I_{0}\left[a_{1}e^{-\left(\frac{u^{2}{}_{1}Q^{2}}{6}\right)}+a_{2}e^{-\left(\frac{u^{2}{}_{2}Q^{2}}{6}\right)}\right].$$ (4)

〈*u* ^2^〉represents the full amplitude of atom dynamics in three dimensions, and atom displacement from the mean position is given by 〈*u* ^2^〉/6.

On the other hand, at low temperatures, protein dynamics are harmonic and can be described as a set of quantitated Einstein harmonic oscillators [40]:

$$\langle u{}^{2}\rangle /3=\frac{h\langle v\rangle }{2k}\left[\text{coth}\frac{h\langle \upsilon \rangle }{2k_{B}T}-1\right]\;,$$ (5)

where *k*_*B*_ is the Bolzmann constant and *h* and 〈*v*〉 are the average frequency and the average force field constant of the oscillators, respectively.

## Results and Discussion

### Effects of the P38G mutation and neutron scattering experiments on protein structure

In order to enable the linking of experimentally measured protein dynamics to biomolecular structure and function, we carried out a characterisation of wtP2 and P2-P38G *in vitro*. These experiments were also aimed at studying any possible changes in protein structure or stability during and after the extensive neutron scattering experiments. Such a detailed sample characterisation allows to conclude, whether differences between samples are caused by large-scale structural re-arrangements, or if they can safely be assumed to result from real dynamical properties.

The human myelin protein P2 has a structure typical for FABPs, where β strands form a 10-stranded barrel covered by an α-helical lid [10,11]. Proline 38 lies at a putative hinge between the barrel and the lid (Fig 1a), and the P38G mutation would intuitively be expected to make the hinge more flexible, perhaps aiding lid opening motions or making the portal region less stable [11]. The crystal structure of P2-P38G (Table 1) indicates that the overall structure of P2 in the crystal state does not change due to the point mutation from an amino acid with the most rigid (Pro) to the one with the most flexible (Gly) backbone. The fatty acid palmitate is bound in the same mode in both forms of P2, and the flexible loops at both ends of the barrel are also in similar conformations. The helical lid is also closed in the mutant protein in the crystal state (Fig 1b).

**Table 1:** Crystallographic data collection and refinement statistics.

Dataset	wtP2 after EINS	P2-P38G
*Data collection*		
Beamline	I911-3 (MAX-Lab)	X12 (DESY)
Unit cell dimensions	a = b = 57.7 Å, c = 100.7 Å	a = b = 64.3 Å, c = 101.4 Å
Space group	P4_1_2_1_2	P4_1_2_1_2
Resolution (Å)^a^	20–1.45 (1.49–1.45)	20–2.12 (2.17–2.12)
R_merge_ (%)	4.9 (147.8)	5.1 (75.6)
<I/σI>	21.9 (1.3)	23.5 (1.7)
Completeness (%)	99.8 (99.9)	97.8 (84.9)
Redundancy	7.0 (6.5)	7.7 (4.2)
CC_1/2_ (%)^b^	100 (40.4)	100 (70.8)
*Refinement*		
R_work/_R_free_ (%)	14.7/18.4	21.9/27.3
RMSD		
Bond lengths (Å)	0.009	0.004
Bond angles (°)	1.3	0.7
Molprobity score (percentile)^c^	1.04 (99th)	0.77 (100th)
Ramachandran most favoured (%)^c^	100	100
Ramachandran outliers (%)^c^	0	0
PDB code	4D6A	4D6B

^a^ The numbers in parentheses refer to the highest-resolution shell.

^b^ CC_1/2_ is defined as the correlation coefficient between two random half-datasets [41]

^c^ Validation was carried out using Molprobity [28]

**Fig 2:**
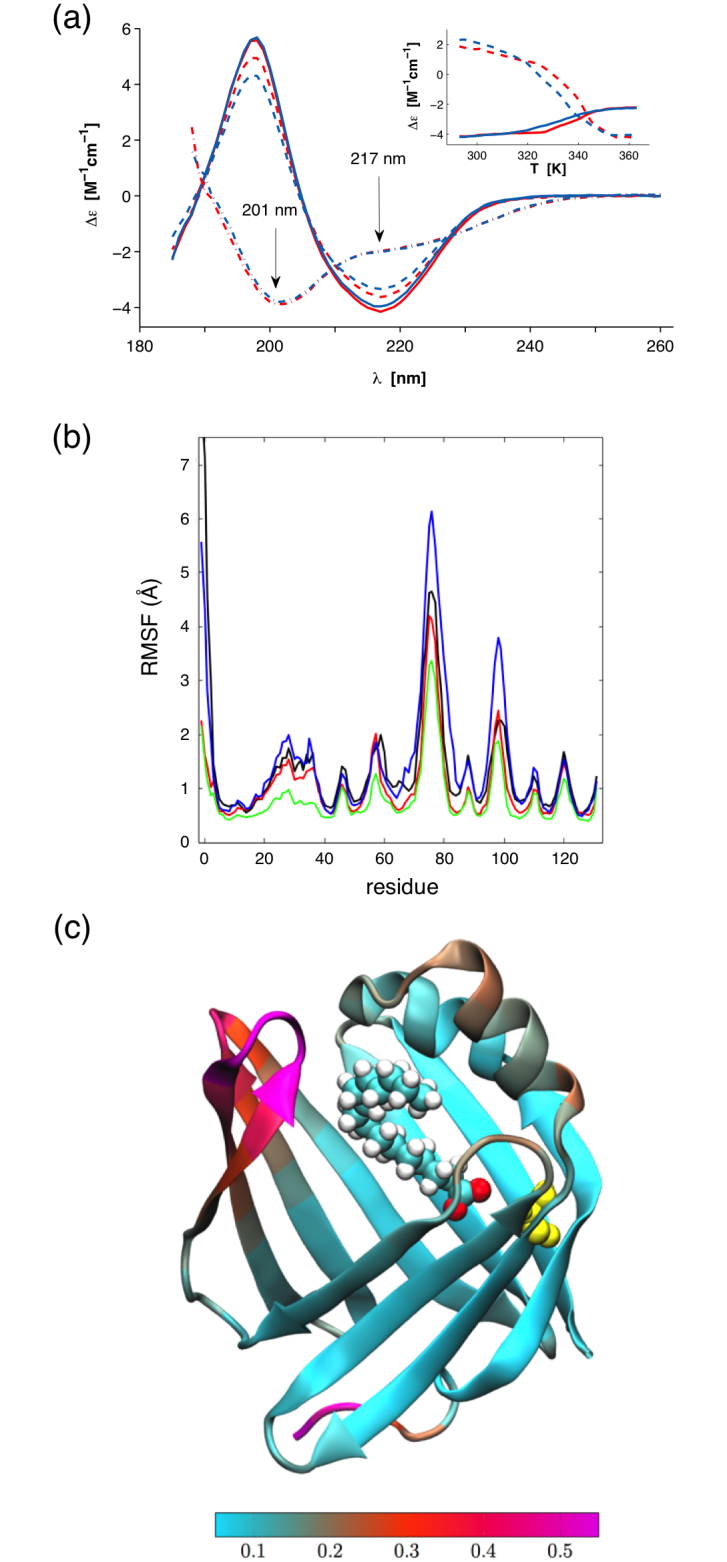
Folding and dynamics of P2. (a) CD spectra of wtP2 (red) and P2-P38G (blue) in solution. Solid lines represent the samples before neutron scattering experiments and dashed lines the samples after these experiments at 293 K. Dashed dotted lines are denatured samples at 363 K. The melting curves at 201 (dashed line) and 217 nm (solid line) as a function of temperature are shown in the inset. (b) Root-mean square fluctuation (RMSF) per residue analyzed for all the four simulated systems from the final 2500 ns of the simulation trajectory. Data for P2 without palmitate are shown in black (wild type) and red (P38G mutant). The results for P2 with palmitate are depicted in green (wild type) and blue (P38G). Large RMSF values represent regions with high flexibility. The most flexible parts of the protein are found at the two loops opposing the lid. (c) A visualisation in VMD [42] of the RMS fluctuations in the P38G mutant with the palmitate chain inside the binding pocket. The yellow color corresponds to the mutated amino acid Gly38. The most flexible parts of the protein are pictured in bright magenta color. The colour bar at the bottom describes the range of RMSF values (light blue for low, violet for high).

wtP2 and P2-P38G have indistinguishable folding in solution as well, as shown by CD spectroscopy (Fig 2a). The melting curves for both proteins have similar trends: complete unfolding happens slowly between 310 and 350 K, the melting temperature being 331 K for wtP2 and 325 K for P2-P38G. This difference of 6 K in temperature stability is also likely to be reflected in the respective dynamics of wtP2 and P2-P38G, indirectly suggesting a more dynamic nature for the mutant protein.

To perform a neutron scattering experiment, the protein samples need to survive harsh conditions that are far from a physiological environment: dialysis to pure water, lyophilisation, rehydration with D_2_O, time-consuming cycles of cooling and heating between 20 K and 310 K, and long neutron beam exposures. Therefore, the P2 protein quality before and after the neutron scattering experiment was analyzed. The P2 secondary structure content did not change, as seen in CD spectra (Fig 2a). To further check for the integrity of the sample during the experiments, wtP2 was resolubilised and crystallised after the neutron scattering experiments, and its crystal structure was refined at high resolution. The structure is essentially identical to that of fresh P2 (Fig 1a), proving that the P2 sample remains stable during the preparation of a hydrated powder and the neutron scattering measurements.

### Simulation of P2 dynamics

As the crystal structures of wild-type and mutant P2 were essentially identical, we looked for differences in their dynamics with computer simulations. In addition to the expected increase in dynamics at the site of the mutation, the entire portal region—which must open, when a fatty acid enters—was expcted to be more flexible in the mutant. This concerns especially the second helix of the helical lid, which we predicted to unfold upon lipid membrane binding [11]. Our earlier simulations, furthermore, suggested effects on the lid dynamics by the bound fatty acid [11]. The effect of the fatty acid was confirmed by the 2.5-μs simulations here, as wtP2 was clearly stabilised by the bound fatty acid. On the other hand, bound ligand appeared to destabilise the portal region in the mutant protein.

The P2 protein is a β barrel with four loops at the top and at the bottom, and a lid consisting of two short α helices connected by a loop. The palmitate ligand resides inside the barrel, with its hydrophobic tail facing the lid. Pro38 lies in a short turn between the end of the second α helix and the subsequent β strand.

P2 crystal structure consists of a total of 132 amino acids. The lid consists of two α helices (16–23 and 27–36) and the loop between them. The loops on the lid side of the protein contain residues 55–58, 74–78, 97–99, and 119–122. The loops at the bottom of the protein are formed by residues 46–47, 65–68, 88–89, and 110–111. Apart from the N and C termini, the rest can be considered to be a β sheet structure, which forms a barrel.

RMS fluctuations characterise the dynamics of the protein (Fig 2b and 2c). The most flexible parts of P2 can be observed to reside on the side of the lid, *i*.*e*. within the portal region. The two loops apposing the lid (residues 74–78 and 97–99) are the most mobile parts of the protein (Fig 2c). Surprisingly, the lid itself does not move as much, as one might expect.

Comparing the different systems to each other, there do not seem to be significant differences in the dynamics of the palmitate-free proteins, though wt-P2 seems to be slightly more flexible than the P38G mutant. However, when palmitate is present, wt-P2 is considerably more stable than either of the palmitate-free proteins. Conversely, P2-P38G with bound palmitate is much more flexible, especially in the two loops apposing the lid. A point mutation at the root of the lid, therefore, renders the lid much more apt to open. This conclusion is supported by observations that the palmitate chain attempted multiple times to escape the barrel of the P38G mutant during the simulation (data not shown).

### Functional assays

**Fig 3:**
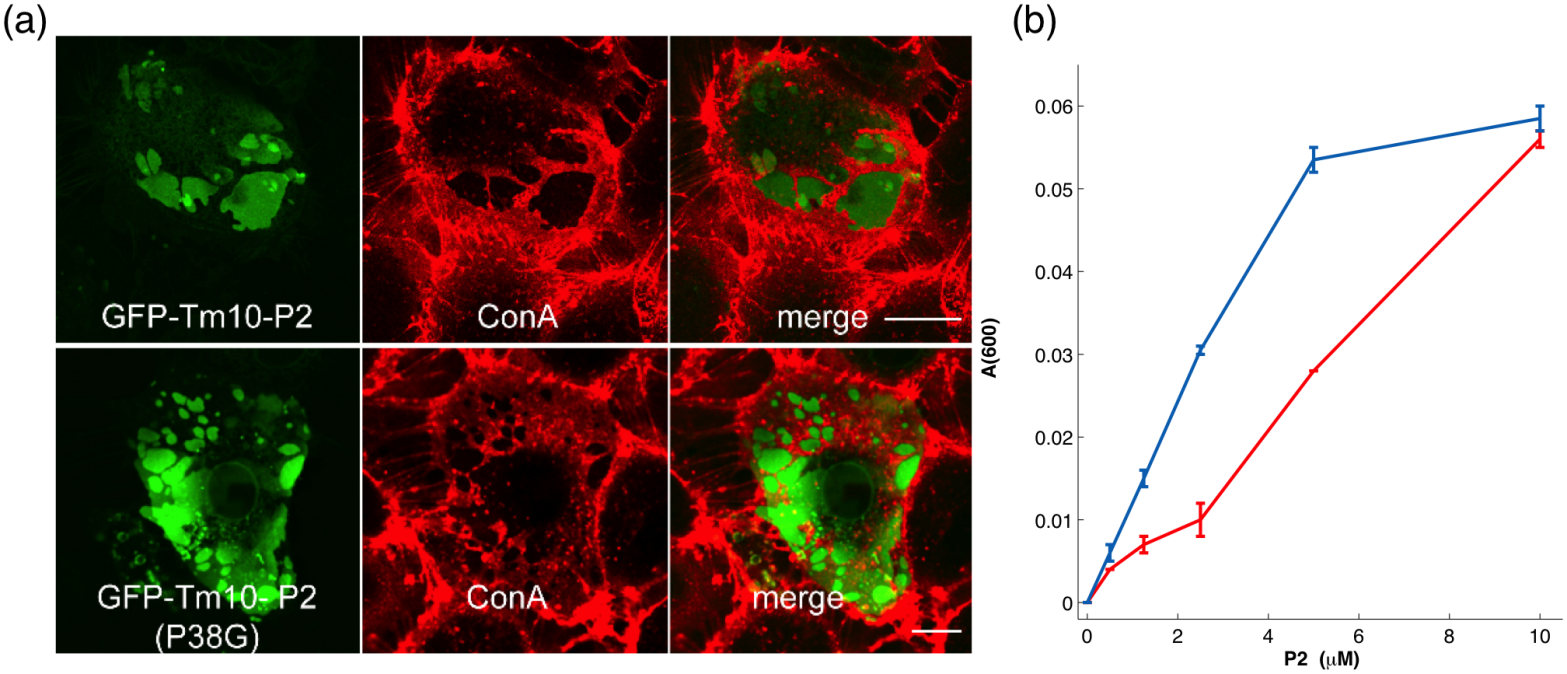
Functional assays. (a) Overexpressed wtP2 (top) and P2-P38G (bottom) both form stacked membrane domains in cell culture. The scale bar is 10 μm. Cell surface glycoproteins (visualised by concanavalin A; red) are depleted from the stacked membrane domains containing fluorescent P2 (green). (b) wtP2 (red) and P2-P38G (blue) both induce vesicle aggregation, which can be observed as increasing turbidity.

P2 is likely to stabilise the myelin structure and stack membrane layers together in compacted PNS myelin [43]. *In vitro*, membrane stacking and stabilisation by P2 have been observed earlier using different methods [11,13,43]. P2 was also shown to stack lipid bilayers in a vesicle aggregation assay, the hydrophobic Leu residue at the tip of the α-helical lid of P2 being crucial for membrane binding [11]. The latter experiment was also carried out for P2-P38G. The P38G mutation in the hinge region actually enhances the lipid stacking property of P2, which can be seen as increasing turbidity of a DMPC/DMPG vesicle solution at low P2 concentrations (Fig 3a).

In cultured Ptk2 cells, the overexpression of wtP2 causes the formation of stacked membrane domains, while the membrane binding-deficient mutant L27D failed to form such domains [11]. P2-P38G induces the formation of similar membrane domains as wtP2, also resulting in the extrusion of cell surface glycoproteins from the affected areas, proving the functionality of this variant also in a cellular environment (Fig 3b). Taking all the structural and functional characterisation together, P2-P38G is functional, and it is also stably folded in the crystal state and in solution, like the wild-type protein. Hence, any differences in mutant *vs*. wild-type protein dynamics will not be caused by large conformational differences between the samples or by misfolding of the mutant protein.

### Elastic incoherent neutron scattering

**Fig 4:**
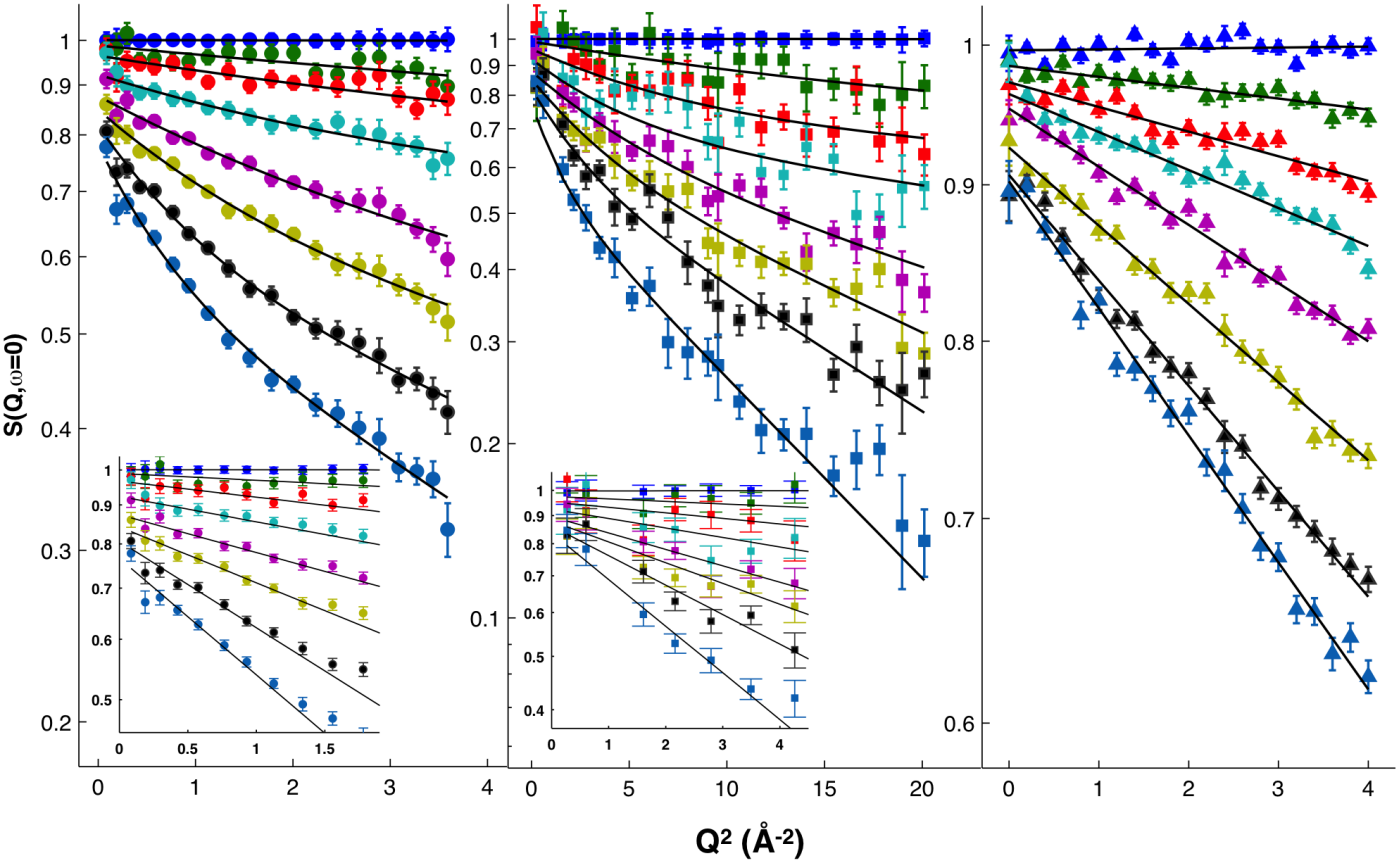
Temperature dependence of the normalised elastic scattering intensities. Data from wtP2 were acquired on IN16 (left), IN13 (middle), and IN6 (right). Data are shown for the temperatures 20, 100, 150, 200, 240, 260, 280, and 300 K (from top to bottom). Solid lines represent the best fitting to IN16 and IN13 data using Eq 4 and to IN6 data using Eq 1. Insets: magnification of the fitting region using Eq 1.

Changes in P2 dynamics upon the introduction of the P38G point mutation were experimentally monitored as a function of sample temperature using EINS. The analysis of IN6 data in terms of a bimodal distribution was the subject of our earlier investigation, showing that the data, unlike those from IN13 and IN16, were best fitted using the Gaussian approximation [23]. The temperature dependence of the elastic scattering intensity, S(Q,ω = 0), of wtP2, normalised to 20 K temperature data, is reported in Fig 4 with fitting using the bimodal distribution model (Eq 4) and the Gaussian model (Eq 1).

The observed protein dynamics depend on the distance and time scales explored. Indeed, data at 200 K clearly show a deviation from a linear behaviour on IN16, which is less enhanced on IN13 in the low-Q region, but more evident at the fully explored Q-interval (Fig 4). This observation is more pronounced at higher temperatures. The deviation is absent in IN6 data, which is a sign of certain motions detectable in the 100 ps—1 ns time scale, indistinguishable within the 10-ps time window of IN6. These motions are likely to be fast methyl group rotations and slower amino acid side chain fluctuations [19,39].

**Fig 5:**
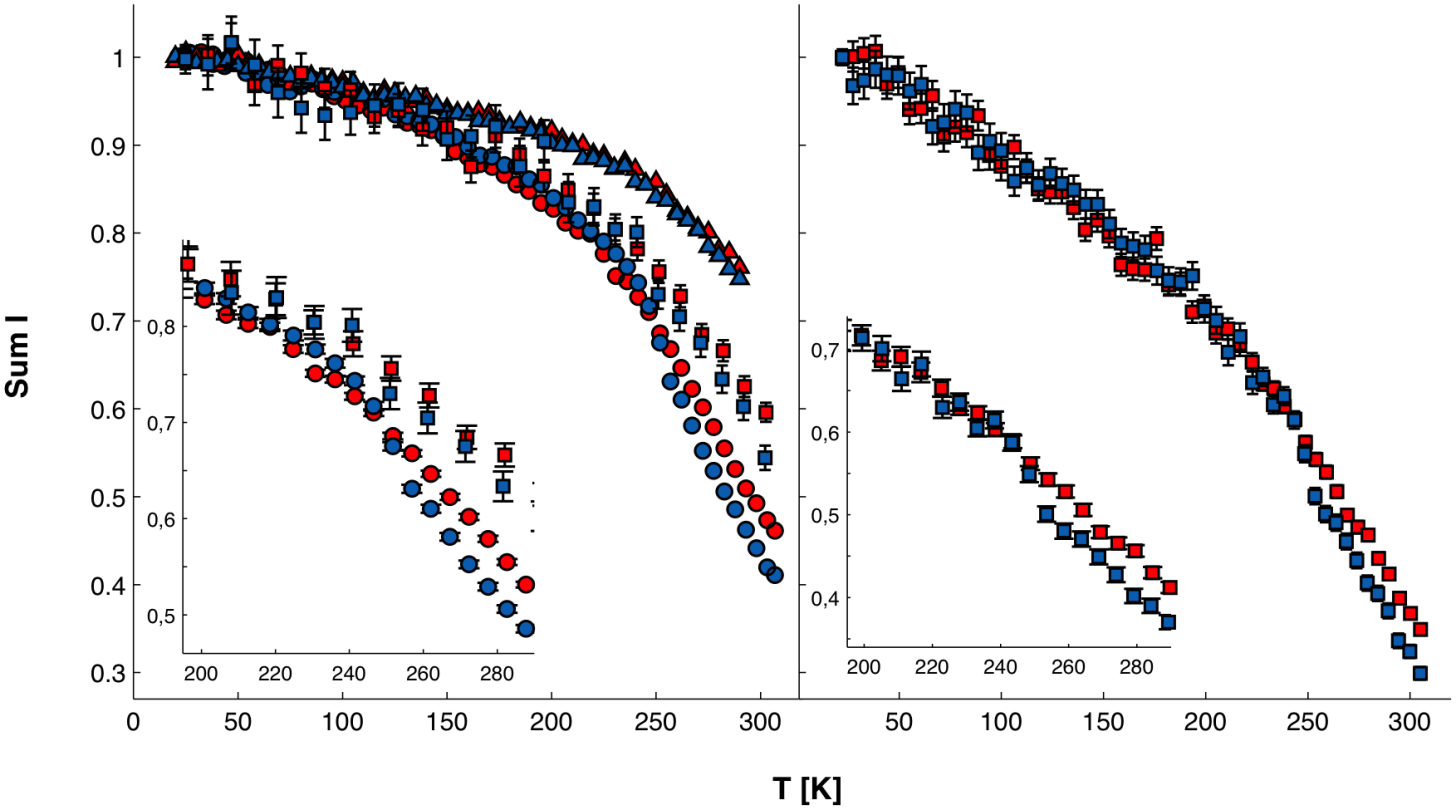
Normalised sums of intensities. Left: Normalised sum over S(Q,ω = 0) at 0 < Q < 2 Å^-1^ on IN6 (triangles), IN13 (squares), and IN16 (circles). Right: Sum over S(Q,ω = 0) at 0 < Q < 4.9 Å^-1^ on IN13. wtP2, red; P2-P38G, blue.

A first insight into protein dynamics from EINS data was obtained from the sum of elastic incoherent scattering intensities over a Q-range as a function of temperature from 20 K to 305 K. The normalised summed elastic scattering intensities of P2 samples from data acquired on IN6, IN13, and IN16, grouped over the Q-range 0.2 < Q < 2 Å^-1^, as well as IN13 data binned over the whole accessible Q-range (0.2 < Q < 4.9 Å^-1^), are shown Fig 5. IN13 data are binned in 10-K steps to improve the statistics.

A deviation from a linear decrease of the summed elastic intensities *vs* T, *i*.*e*. a change in the slope, is a clear sign of the activation of a motion falling within the time scale accessible through the energy resolution of the used instrument. For wtP2, in IN6 data, the first change in the slope can be seen at 180 K, in IN13 data at 150 K, and in IN16 data at 130 K. A second, now instrument-independent, transition is seen at 240 K, above which the elastic signal is reduced for P2-P38G compared to wtP2 on all three instruments. In IN16 data, P2-P38G has a very rapid decrease in elastic signal at 240 K, but from 245 K on, the elastic signal continues with the same slope as wtP2. The same step can be seen on IN13, when the elastic signal over the full Q-range 0 < Q < 4.9 Å^-1^ is taken into account (Fig 5, right panel).

**Fig 6:**
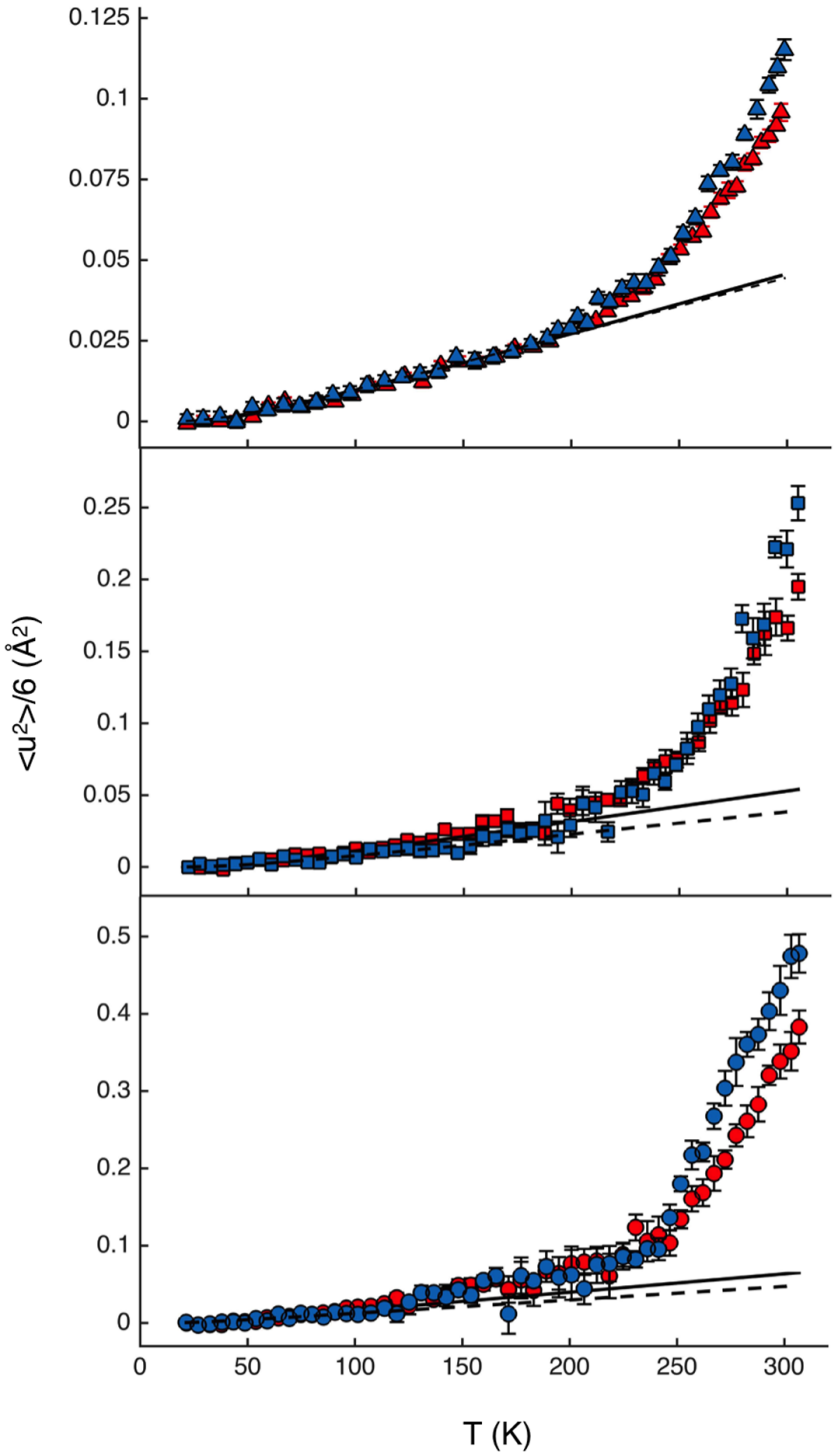
Temperature dependence of the MSD. Data from wtP2 (red) and P2-P38G (blue) were evaluated from the fit of data acquired on IN6 (top), IN13 (middle), and IN16 (bottom) using Eq 1. The low-temperature fits using the Einstein harmonic oscillator function (Eq 5) are shown as solid (wtP2) and dashed lines (P2-P38G).

Gaussian approximation was used to estimate the total MSD of P2 samples on different time scales. IN6 data were fitted over the full Q-range to Q^2^ = 4 Å^-2^ (Fig 4, right panel), IN13 data to Q^2^ = 3.5 Å^-2^ (Fig 4, middle panel, inset), and IN16 data to Q^2^ = 0.77 Å^-2^ (Fig 4, left panel, inset) using Eq 1. The Q^2^ region for Gaussian validity is instrument-dependent, being strictly linked to the MSD. Indeed, the Gaussian approximation is valid in the low-Q region, where log(S(Q,ω = 0)) is linear as a function of Q^2^, and, generally, when *Q* ^2^〈u^2^〉≤ 2 [17]. However, it depends heavily on the geometry of the motion. In a protein, the dynamics of an atom can be defined as localised motions within an ellipsoidal shape with axes 1:1:1.7 [44]. For elliptically moving atoms in proteins, the Gaussian approximation is valid to $\sqrt{Q^{2}\langle u^{2}\rangle }\approx 3$ [45]. The determined averaged MSDs are presented in Fig 6. The Gaussian approximation is valid up to the highest measured temperatures (305 K) on IN6, IN13, and IN16, where $\sqrt{Q^{2}\langle u^{2}\rangle }$ of P2-P38G reaches the maximum values 1.6, 2.3, and 2.0, respectively.

At low temperatures, proteins vibrate locally, and their dynamics can be described using the Einstein harmonic oscillator function [40]. The estimated MSDs of IN6 and IN13 data were fitted from 20 to 150 K, and the MSD of IN16 data to 130 K, using Eq 5 (Fig 6, Table 2). The average harmonic vibration for both wtP2 and P2-P38G varies within error bars, reaching 〈u^2^〉 = 0.05 Å^2^ at 300 K on all three instruments. The fitted average oscillation frequencies *v* are around 2.5 ps^-1^ for IN6 and IN13 data, and approximately half of that for IN16 data. The estimated average force field constants (*k*) are 3–4 N/m. While the values of both *k* and *v* are comparable to those estimated for artificial membranes [40], no significant difference can be observed between wtP2 and P2-P38G.

**Table 2:** EINS data fitting parameters.

Instrument	Sample	wtP2		P2-P38G	
Gaussian approximation					
IN6	k [N/m]	3.7±0.3		3.7±0.2	
	ν [ps^-1^]	2.5±0.4		2.5±0.3	
IN13	k [N/m]	3.2±0.4		4.3±0.8	
	ν [ps^-1^]	2.5±0.4		2.7±0.6	
IN16	k [N/m]	2.9±0.4		3.9±0.6	
	ν [ps^-1^]	1.5±0.4		1.3±0.4	
Bimodal distribution model					
		H_methyl_	H_non-methyl_	H_methyl_	H_non-methyl_
IN13	k [N/m]	2.0±0.5	5±1	2.1±0.8	6±2
	ν [ps^-1^]	2±1	3±1	2±1	3±1
IN16	k [N/m]	0.6±0.1	20±2	0.6±0.1	25±4
	ν [ps^-1^]	2.3±0.7	2.8±0.5	2.1±0.9	2.6±0.7

The first deviation of MSD from harmonic oscillation occurring at 120–180 K is thought to be the hydration-independent thermal activation of methyl proton rotations [46,47]. The methyl group activation (MGA) temperature has been shown to be instrument resolution-dependent [39]. The frequency of methyl group rotation depends on the amino acid side chain and its chemical environment [48] and increases as a function of temperature. Therefore, methyl rotations become observable at lower temperatures on instruments with the narrowest energy resolutions and longer time scales. In particular, the MGA temperatures of IN6, IN13, and IN16 have been determined as 180 K, 150 K, and 110 K, respectively, for a hydrated alanine polypeptide and albumin [39]. These temperatures are in good agreement with our P2 data, where MSD values deviate from the purely harmonic behavior fit at the MGA temperatures (Fig 6).

The transition at 240 K is generally called the protein dynamical transition (PDT). The origin of PDT has been under debate, but it is generally agreed that PDT is not dependent on instrument resolution but strongly depends on protein hydration [19,39,49]. In data from P2 samples, P2-P38G is systematically more dynamic than wtP2 above the PDT at all three measured energy resolutions (Fig 6). A steeper slope of MSD at high temperatures indicates increasing protein flexibility and softening of the protein [20].

Within a protein, atom dynamics do not correspond to free diffusion, but instead, all the atoms and their fluctuations are connected to their neighbouring atoms. The degree of freedom of hydrogen dynamics depends on the location of the hydrogen atom in the amino acid, such that the outermost side-chain hydrogen atoms are in general more mobile. The side chain dynamics depend on the length and polarity of the amino acid residue, as well as on the chemical environment of the side chain, which in turn is determined by the folding of the entire protein chain. Accordingly, an unfolded polypeptide chain is more flexible than a tightly folded globular protein [50,51].

A model that could univocally describe protein dynamics does not exist. Several different models have been introduced [39,52,53] to elucidate the main dynamical processes occurring in a protein molecule. Here, in the bimodal model chosen by us, the hydrogen atoms of P2 were divided into methyl and non-methyl hydrogens, with the population fractions a_1_ = 0.26 (methyl) and a_2_ = 0.74 (non-methyl), corresponding to the calculated number of non-exchangeable hydrogen atoms in the P2 protein. It should be noted that these fractions are expected to be temperature-independent. We also tested the fitting procedure allowing the population fractions to vary (data not shown); a slight, negligible, effect on the observed methyl group fraction can be seen with increasing temperature, and the fractions converge to a_1_ = 0.26 (wt-P2) and a_1_ = 0.32 (P2-P38G) at room temperature (293 K)—very close to the values calculated based on chemical composition. In general, protein main-chain atoms are the most rigid, and the hydrogen atoms of long amino acid side chains furthest away from the main chain have the largest degree of freedom. Protein folding into secondary, tertiary, and quaternary structures, as well as protein interactions with binding partners, prohibits side chain rotations, reducing the possible side chain conformations [54,55]. Hydrogen atoms in methyl groups present an exception, as they have a high degree of freedom of rotation. Hydrogen atoms in a methyl group are chemically equivalent, and being uncharged, methyl groups do not form classical polar interactions [55]. Hence, methyl group dynamics are expected to provide the main contribution to global protein hydrogen dynamics.

Using a bimodal model for EINS data analysis, a distinction has previously been made between hydrogen atoms on the protein surface and in the core [21]. Unlike other proteins studied earlier by incoherent neutron scattering, P2 is a hollow barrel with a large cavity inside. Therefore, most of the side chains of P2 are in contact with solvent or the ligand inside the cavity (Fig 1c). The water molecules surrounding the P2 protein and within its internal cavity at cryo temperatures in the crystal state are shown in Fig 1d. According to protein dynamics simulations [56], the location of a residue—on the surface or in the core—has no remarkable effect on residue rigidity. Therefore, the distinction of hydrogen atoms between core and surface groups is not needed, and classification as methyl and non-methyl groups is justified in the current analysis.

**Fig 7:**
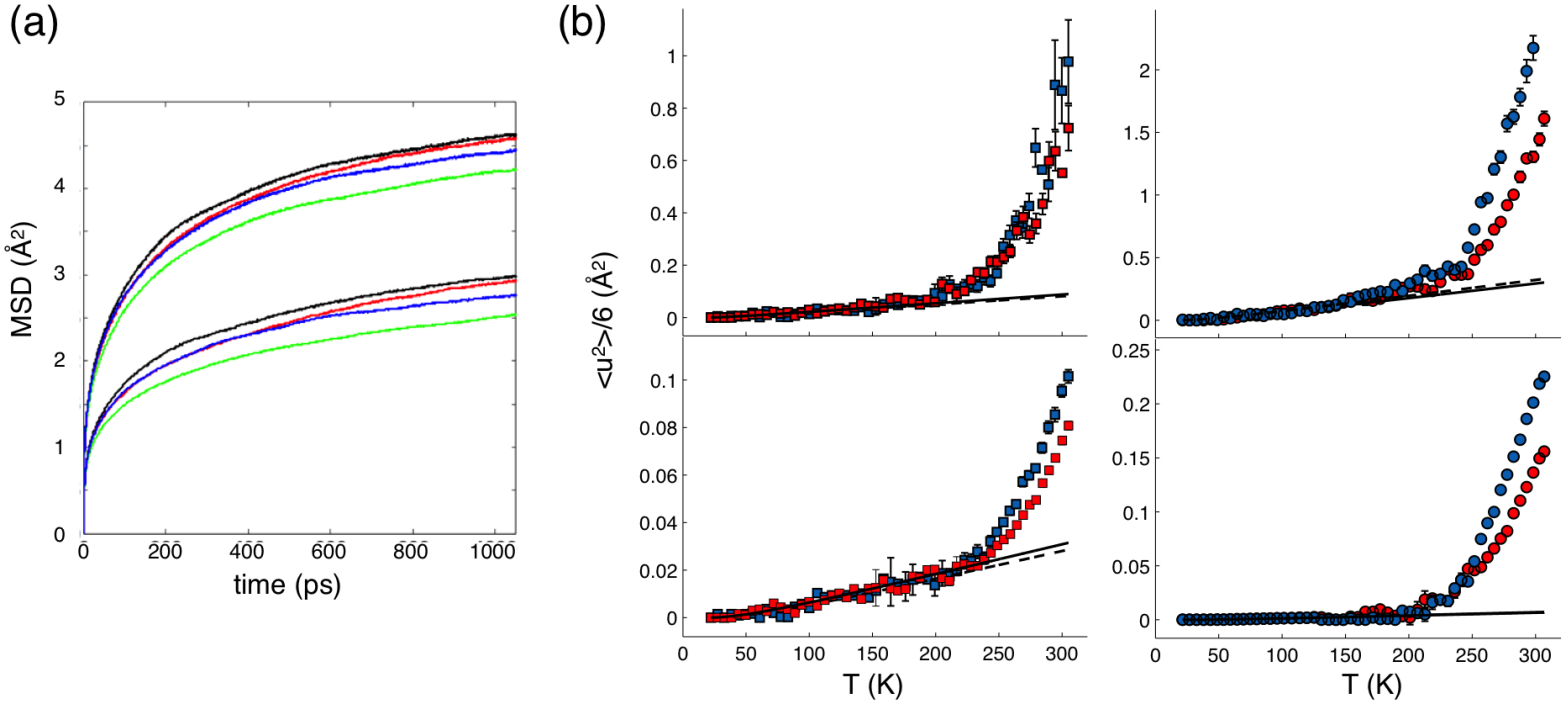
Simulated and experimental hydrogen MSDs. (a) The averaged MSD of i) hydrogens in CH_3_-groups (top group of curves with higher MSD values) and ii) hydrogens in CH_2_- and CH-groups (bottom group with lower MSD values) for all the four systems studied by MD simulations. Data for P2 without palmitate are shown in black (wild type) and red (P38G mutant). The results for P2 with palmitate are depicted in green (wild type) and blue (P38G). (b) Experimentally determined MSD contributions from methyl (upper panels) and non-methyl hydrogens (lower panels) for wtP2 (red) and P2-P38G (blue) using the bimodal model. Left: IN13; right: IN16. Solid (wtP2) and dashed (P2-P38G) lines represent the low-temperature harmonic vibrational contributions.

Molecular dynamics trajectories were further analysed to validate the experimental model and data. MSDs were used to quantify the dynamics of the protein hydrogens separately for hydrogens in CH_3_-groups, and for hydrogens in CH_2_- and CH-groups. Fig 7a depicts that the methyl hydrogens are much more mobile than other hydrogens both in terms of the distance they travel (magnitude of MSD), and in their rate of diffusive motion (tangents of the MSD curves). The hydrogens in the palmitated proteins are found to be more constrained than in their palmitate-free counterparts. In palmitate-free proteins, the hydrogens in wt-P2 are slightly more mobile than in P2-P38G. Conversely, in the proteins with bound palmitate, the hydrogens of the P38G mutant are more mobile than in wt-P2. The results confirm earlier indications of effects of bound fatty acid on P2 dynamics [11], and they justify the division of P2 hydrogens into methyl and non-methyl groups for the analysis of EINS data.

Similarly to the MSD defined by Gaussian approximation on IN13 and IN16, there is no visible difference between wtP2 and P2-P38G dynamics below 220 K in non-methyl hydrogen atoms, when the data are analyzed using the bimodal distribution model (Eq 4; Fig 7b, bottom). As expected, no MGA can be seen for non-methyl hydrogen atoms, and MSD follows the fitted harmonic oscillation contribution up to 200 K. The non-methyl hydrogen contribution seen on IN13 is close to the total dynamics seen on IN6 (Fig 6), with similar fitting parameters for low-temperature fluctuations (Table 2). On IN16, the non-methyl hydrogen contribution is very small below 200 K, resulting in force constant *k* values higher than 10 N/m. The short and fast fluctuations of non-methyl hydrogens occurring at low temperatures are probably out of the instrument resolution window and thus cannot be detected on IN16. Overall, there is no difference in the non-methyl hydrogen MSD values between wtP2 and P2-P38G samples obtained on IN13 and IN16 below 240 K, above which P2-P38G is more dynamic and its MSD higher.

The methyl proton contributions seen on IN13 and IN16 oscillate at low temperatures with frequencies *v* of 2 ps^-1^ (Table 2), and estimated force constants *k* are 0.6 N/m for IN16 time scale vibrations and approximately three times higher for the dynamics seen on IN13. The MSD determined on IN13 faces MGA at 150 K, after which dynamics of the wild-type and mutant protein increase similarly up to the 240 K PDT temperature (Fig 7, left, top). On IN16, the MGA occurs at 130 K. Differently to IN13 data, at IN16 resolution, P2-P38G is more dynamic than wtP2 already between the MGA and PDT. In IN13 and IN16 data, the MSD of methyl hydrogens of P2-P38G increases more rapidly at PDT (240 K). This is in good agreement with the clear change in the slope of the sum of elastic intensities (Fig 5) at 240 K.

The total MSD of P2 is dominated by dynamics of the methyl hydrogens (Fig 7). Methyl groups are nonpolar moieties that are located outermost in amino acid residues, having less neighbouring atoms and more space to rotate freely. In P2, methyl groups are scattered throughout the protein (Fig 1c) and often have contact to the solvent.

The point mutation P38G in the hinge between the barrel and lid of P2 does not affect P2 folding (Fig 1a) or render it inactive (Fig 3a), but it increases the total flexibility of the protein (Fig 6). The dynamics experimentally observed above the PDT may be related to such functional dynamics of P2. Using EINS, only averaged total dynamics can be observed, and additional experiments and molecular dynamics simulations will be needed for understanding the results.

Inside the P2 β barrel, there is a bound fatty acid from the bacterial expression host. While P2, although being a folded protein, has no clearly defined hydrophobic core, the bound ligand could provide such a core, possibly playing a role in P2 stability. Could the differences between wild-type and mutated forms of P2 be partially related to the bound fatty acid? Firstly, the dynamics contributed by the fatty acid can be ignored from data analysis, because its hydrogens correspond to only 3% of the protein hydrogen atoms; the scattering from the fatty acid would be hidden by the much larger protein dynamics contribution. Secondly, an absence of bound fatty acid could increase the amount of water inside the barrel, which in turn could change the protein dynamics. In the crystal structures, though, the fatty acid is bound, and its interactions with the protein are similar. Possible differences in fatty acid binding between the two samples could lead to differential flexibility of the portal region. Thus, also in this situation, the observed increased dynamics of P2-P38G would be explained with an increasing flexibility of the hinge that allows the lid to move more freely in the mutant molecule. Intriguingly, our extended MD simulations revealed that while the wild-type protein was stabilised by bound fatty acid, the mutant actually became more dynamic, especially within the portal region. The P2-P38G simulation trajectory reveals that the tail of the fatty acid is extended throughout the portal region during the simulation, which would mimic eventual dissociation of the ligand; this is expected to happen, when P2 binds to a membrane surface [11]. The simulations, thus, both confirmed the increased dynamics of P2-P38G and justified the use of the bimodal model in EINS data analysis.

## Conclusions

In order to fully understand structure-function relationships in proteins, an insight into protein dynamics is crucial. EINS allows the inspection of changes in total protein dynamics as a function of an external parameter, such as temperature. We used neutron scattering and complementary methods to study the link between P2 protein dynamics and its structure-function relationships. P2 is a structural component of myelin, and its function is most likely related to its membrane binding capacity. The P38G mutation increased the lipid membrane stacking capacity of P2 *in vitro*, and the mutated protein was also correctly folded and active in cells. However, neutron scattering indicated that the P38G mutant is more dynamic than the wild-type protein, especially on a slow 1-ns time scale. The fact that the mutation causes significantly increased dynamics of the portal area in P2 enabled us to use EINS in this study to experimentally observe differential dynamics of a wild-type and a mutant protein. Earlier, we have shown that binding to membranes induces changes in P2 structure, mainly partial unfolding of α-helices of the lid [11]. The increased membrane binding activity of P2-P38G could be explained by increasing protein flexibility and an enhanced ability to interact with lipids. While EINS allows the inspection of the total dynamics, it is limited with respect to analysis of the origin and type of dynamics. Therefore, other methods, such as quasielastic neutron scattering and atomistic simulations on membrane binding, can be used in the future to provide more detailed information about functionally relevant protein dynamics in P2.
